# Smartphone-based patient-driven teledentistry for postoperative monitoring: a pilot study

**DOI:** 10.1007/s00784-026-06840-9

**Published:** 2026-03-26

**Authors:** Benjamin Pasztor, Franz J Strauss, Ronald E Jung, Marc Balmer, Tim Joda, Riccardo D Kraus

**Affiliations:** 1 Clinic of Reconstructive Dentistry, Center for Dental Medicine, University of Zurich, Zurich, Switzerland; 2 Universidad Autonoma de Chile, Santiago, Chile; 3 Department of Reconstructive Dentistry, University Center for Dental Medicine, University of Basel, Basel, Switzerland

**Keywords:** Teledentistry, Oral surgery, Wound healing, Remote monitoring, Intraoral camera

## Abstract

**Purpose:**

This pilot study evaluated whether the use of an intraoral camera improves the accuracy of remote postoperative wound-healing assessment after oral surgery compared to a phone consultation alone. The primary outcome was the correct identification of cases requiring further intervention such as a medication change or an in-person visit.

**Methods:**

Eleven patients undergoing oral surgery were assessed 4–6 days postoperatively using four remote modalities: phone consultation (PC), patient-generated snapshots (SS), patient-generated video footage (VF) and clinician-guided live video call (LC). For each method, assessments were performed independently by the operating clinician and by a second independent dentist, who had no prior knowledge of the patient, using standardized questionnaires developed for this study. A physical visit (PV) served as the reference standard. Accuracy, rates of non-assessable outcomes and inter-examiner agreement (Cohen’s kappa, or prevalence-adjusted bias-adjusted κ when required) were calculated. Performance across modalities was compared using McNemar tests. Clinicians also rated the perceived usefulness and preference and image quality was assessed for smartphone-based intraoral camera (IOC) modalities.

**Results:**

No patient required an intervention at the PV. Remote assessments incorrectly suggested the need for intervention in three instances, all occurring during phone-based or live video call evaluations. Accuracy varied across modalities. Correct identification of cases needing intervention was highest for SS and VF (21/22; 95.5%), followed by PC and LC (19/22; 86.4%). Snapshots showed the lowest overall accuracy across wound-healing parameters, largely due to poor image quality (47% diagnostically sufficient). Live video calls yielded the highest proportion of diagnostically adequate images (72.7%) and were the most preferred modality among clinicians. Across all modalities, assessments by the operating clinician more closely matched PV findings than those made by the independent dentist.

**Conclusions:**

In this pilot study, smartphone-based intraoral camera methods did not outperform simple phone consultations in identifying postoperative cases requiring intervention after oral surgery. This finding likely reflects the limited statistical power rather than a true absence of differences between methods. However, live video calls were preferred by clinicians and provided the most clinically useful images.

**Clinical relevance:**

Remote postoperative assessment in oral surgery is feasible but reliability depends largely on image quality and clinician’s familiarity with the patient. Live video-guided assessements may offer practical advantages and represent a promising approach for future teledentistry applications

**Supplementary Information:**

The online version contains supplementary material available at https://doi.org/10.1007/s00784-026-06840-9.

## Introduction

Remote consultation and patient triage have been implemented in medicine since the late 20th century [1]. This allows patients to be screened without an in-person visit thereby reducing unnecessary hospital visits and improving access to care, especially in underserved areas. In contrast, the adoption of such approaches in dentistry has been relatively slow [2]. Incorporating telecommunication technologies into dental care, commonly referred as teledentistry, offers the potential to enhance access, enable early diagnosis, improve patient education, and optimize practice efficiency. These benefits may contribute to reducing health inequalities while providing a more cost-effective and efficient way of assessing pre- and post-operative parameters [3–5]. 

The COVID-19 pandemic indisputably has been a major driving force for the wider implementation and acceptance of teledentistry [6]. For teledentistry to achieve lasting impact beyond the pandemic, however, several barriers must be addressed. Insufficient infrastructure, particularly unreliable internet connectivity in suburban and rural areas has been a major obstacle. With the increasing availability of *5G* networks, faster and more reliable data transfer is becoming feasible [7]. From a legal and regulatory standpoint, robust frameworks are required to safeguard patient privacy and data security given the sensitive nature of transmitted health information [5, 8]. Further challenges include limited familiarity and acceptance among patients, especially older populations, and to some extent dental practitioners themselves, with the technology and resources needed to integrate TD into routine care [9]. 

Among dental specialties, oral surgery appears particularly well suited for TD applications. Recent studies, have evaluated the use of TD for post-operative care after third molar and implant surgery [10, 11]. Remote monitoring of wound healing may enable early detection of complications potentially reducing post-operative problems and, in severe cases, preventing hospitalizations [10]. However, evidence regarding the accuracy of remote assessment systems of teledentistry in detecting wound-healing disturbances and complications remains limited and the optimal modality, whether voice calls, imaging, or a combination of both, has yet to be established [11]. 

Therefore, the aim of this study was, to test whether the use of an intraoral camera for remote wound-healing assessment after oral surgery improves the detection of cases requiring intervention compared to assessment based solely on phone consultation. The primary outcome was the accurate identification of patients needing an intervention such as prescription of medication (e.g. antibiotics) or an in-person visit. The null hypothesis stated that there is no difference in benefit between an evaluation performed using an intraoral camera and an evaluation conducted via a simple phone call.

## Materials and methods

The present pilot study was performed at the Clinic of Reconstructive Dentistry, Center for Dental Medicine, University of Zürich, Switzerland, and approved by the Cantonal Ethics Committee of Zurich (Switzerland) on the 11th of July, 2023 (KEK-ZH-Nr. 2023 − 00142).

### Study population

A total of 11 patients scheduled for surgical interventions recruited. Each patient agreed to attend an additional follow-up appointment 4–6 days post-operatively. This should simulate a remote assessment, which the patient could undergo prior to the standard postoperative visit scheduled 7 days after surgery, including suture removal.

### Study design and modalities

**Fig. 1:**
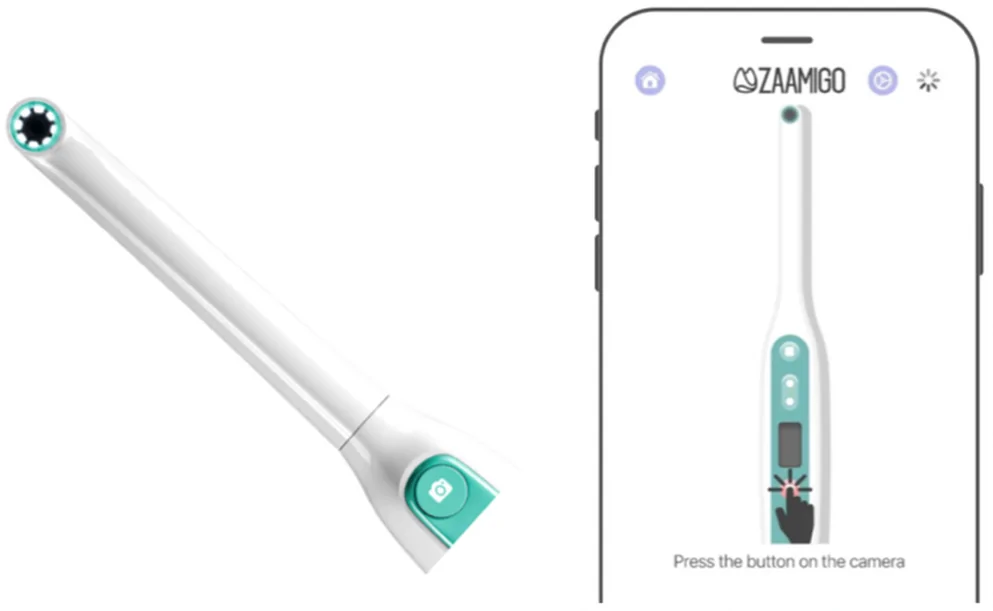
Zaamigo intraoral camera and mobile application interface

To simulate a remote situation, the patients and clinicians were placed in separate rooms. Two examiners participated: an independent dentist (D), unfamiliar with the patient and surgery and the operator (O), who had performed the surgical intervention. Both clinicians sequentially evaluated the patient using four communication modalities:

1. *Phone call (PC)*: Each clinician conducted a phone consultation.
2. *Snapshots (SS) and video footage (VF)*: Patients were provided with an intraoral camera (*Zaamigo*^*®*^,* Zaamigo AG*,* Zurich*,* Switzerland*, Fig. 1) connected to a smartphone (*iPhone 8*,* model MQ7C2ZD/A*,* Apple Inc.*,* Cupertino*,* USA)* via the Zaamigo^®^ application (*Zaamigo AG*,* Zurich*,* Switzerland).* Without staff assistance, patients were instructed to capture three snapshots (SS) and one 15- to 20-second video footage (VF) of the surgical site, simulating a home setting.
3. *Live video call (LC)*: Patients engaged in a live video consultation with the clinician, who guided intraoral camera positioning.

**Fig. 2:**
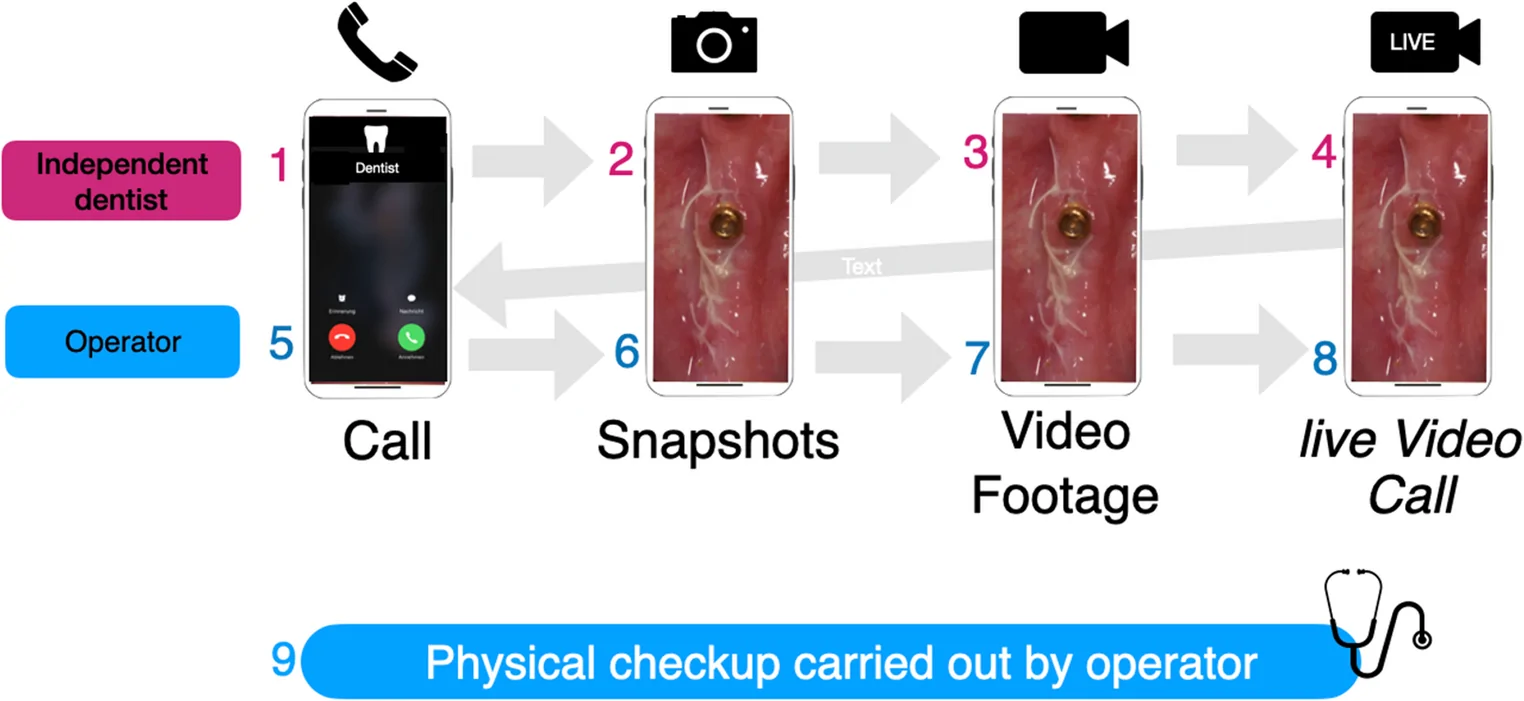
Schematic representation of the study design workflow

Finally, the operator conducted alone a standard physical visit (PV). Figure 2 provides a schematic representation of the study design workflow.

### Questionnaires

For each modality, clinicians completed standardized questionnaires developed for this study. During PC, patients rated discomfort (0 = none, 10 = severe discomfort) related to pain, eating difficulties, and general daily impairment. Additional questions addressed mouth-opening limitations and other symptoms.

Across all modalities, clinicians assessed:

- wound healing on a 1–5 scale (1 = very good, 5 = impaired),
- presence of bleeding, intra- and extraoral swelling (swelling (IO), swelling (EO)), as well as intra- and extraoral hematoma (hematoma (IO), hematoma (EO)).
- flap closure (complete/ incomplete).
- presence of a fine fibrin line or fibrin clot, necrosis, suppuration, and tissue coloration (redder, whiter, or similar to the surrounding tissue) [12].

Assessments of flap closure, fibrin, necrosis, suppuration, and tissue colour were performed exclusively with IOC (SS, VF, LC) and PV. Extraoral swelling and hematoma were assessed only by PC, LC, and PV.

### Image quality

The quality of the images obtained from SS, VF, and LC was assessed using a standardized checklist (“Yes” or “No” responses for appropriateness, exposure (under- or overexposed), focus, stability, correct positioning, and adequacy for clinical assessment.

### Clinician preferences

Examiners determined whether a physical visit was necessary before suture removal or whether an intervention (e.g., new medication) was required (primary outcome). In addition, they ranked the usefulness and personal preference for the four remote assessment modalities (1 = most useful/preferred modality, 4 = least useful/preferred), with scores summed separately for usefulness and preference.

Remote assessments (PC, SS, VF, LC) were compared with PV findings. Assessments were categorized as correct, incorrect, or non-assessable. Percentages were calculated for each category.

### Statistical analysis

Descriptive statistics were performed for all variables (nominal and ordinal), with results expressed as absolute and relative frequencies, and cross-tables were used to compare agreement between examiners. Inferential analyses included concordance testing between examiners (D vs. O) using Cohen’s kappa; for ordinal variables (wound healing, usefulness, preference) a linearly weighted kappa was calculated, and 95% confidence intervals were estimated. Because simple percentage agreement is dependent on marginal distributions and may be misleading, kappa values were used to account for agreement beyond chance. Kappa values were interpreted as follows: 1 = perfect agreement, 0 = no improvement beyond chance, and < 0 = worse than chance. When marginal distributions were extremely unbalanced and kappa was affected by Feinstein’s paradox, the prevalence-adjusted bias-adjusted kappa (PABAK) was applied. McNemar’s test was used to assess symmetry for paired categorical data, comparing the proportion of correct assessments across modalities (PC, SS, VF, LC). The significance level was set at α = 0.05.

## Results

In total 11 patients were assessed after an oral surgical intervention. Seven patients underwent implant surgery, one patient had wisdom tooth surgery and three patients had a soft tissue augmentation procedure.

**Fig. 3:**
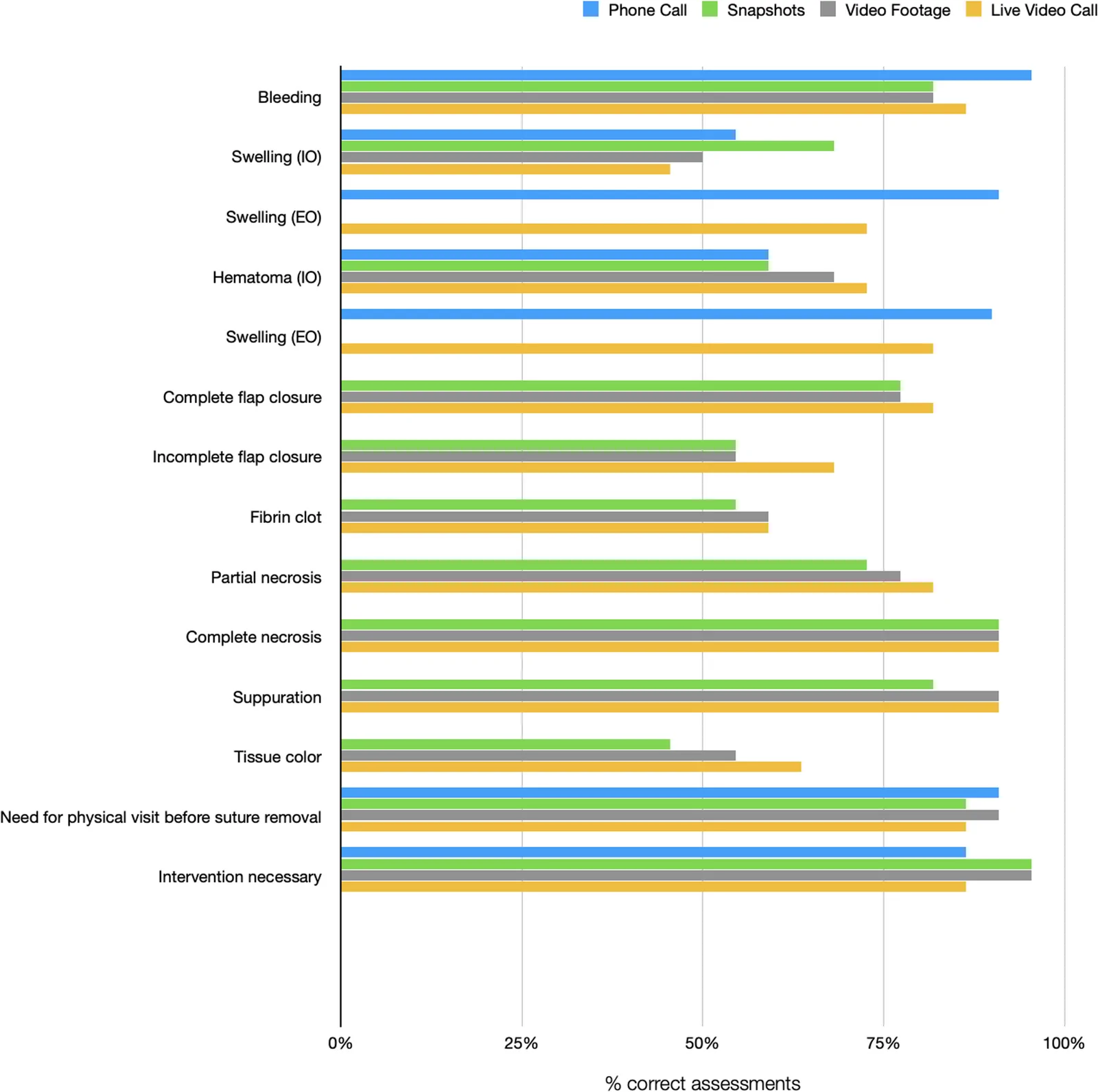
Comparison of diagnostic accuracy across all remote modalities

All remote assessments were compared with the physical visit (PV), which served as the reference standard. A summary of correct (“+”), incorrect (“–”), and non-assessable (NA) evaluations for each modality is provided in Table 1. The results of all parameters are graphically shown in Fig. 3.

**Table 1:** Assessment summary per modality ( “+” = Correct Assessment; NA = Non Assessable; “-“ = Incorrect Assessment)

Phone Call (PC)				Video Footage (VF)			
***+***	70.50%	Operator	69.70%	***+***	69.50%	Operator	74.00%
		Independent dentist	71.20%			Independent dentist	64.90%
***NA***	4.60%	Operator	6.10%	***NA***	7.00%	Operator	9.90%
		Independent dentist	3.00%			Independent dentist	4.10%
***-***	25.00%	Operator	24.20%	***-***	23.20%	Operator	0.20%
		Independent dentist	25.80%			Independent dentist	30.20%
**Snapshots (SS)**				**Live Video Call (LC)**			
***+***	66.10%	Operator	68.20%	***+***	73.80%	Operator	82.20%
		Independent dentist	64.00%			Independent dentist	65.40%
***NA***	9.50%	Operator	14.00%	***NA***	6.30%	Operator	5.60%
		Independent dentist	5.00%			Independent dentist	7.00%
***-***	24.40%	Operator	19.40%	***-***	17.50%	Operator	11.50%
		Independent dentist	29.30%			Independent dentist	23.40%

### Key findings

The assessments which were performed after the various surgeries showed moderate to high agreement with physical visits. Diagnostic performance varied by modality and clinical parameter (Fig. 3): necrosis, suppuration and bleeding were generally correctly identified across all modalities with a particular accuracy in video-based formats. On the other hand, correct assessment of flap closure and intraoral swelling was less frequent.

The need for a physical visit or intervention was determined very accurately across all modalities and no statistical differences were detected between them.

### Comparison of modalities

Assessments carried out through recorded video footage (VF and LC) achieved the highest agreement rates for complications such as necrosis and suppuration (up to 90.9%). Static image-based assessments (SN) performed better when assessing intraoral swelling and significantly outperformed LCs for this parameter (*p* = .016). Also compared to VF assessments there was a detectable trend for superiority (*p* = .063). Assessments performed by PC, although limited, showed very high reliability for detecting bleeding (95.5% correct assessments with the PV as a baseline). Simultaneously the PC modality produced more non-assessable ratings for visually dependent parameters such as general wound healing.

### Analysis of necessity for a physical visit and necessity for an intervention

#### Need for physical visit

Correct determinations of whether an in-person visit was required were highest for both PC and VF (20 of 22; 90.9%), followed by LC (19 of 22; 86.4%) and SS (18 of 22; 81.8%).

The only non-assessable judgment occurred in the LC modality (1 of 22; 4.5%).

No statistically significant differences were detected among modalities.

#### Necessity for intervention

Correct assessments regarding the need for an intervention were highest for SS and VF (21 of 22; 95.5%), followed by PC and LC (both 19 of 22; 86.4%). Non-assessable ratings occurred only during LC (2 of 22; 9.1%). However, these differences were not statistically significant.

During the PV, no interventions were deemed necessary. However, three remote assessments incorrectly indicated a need for intervention:

- *Subject 3*: Operator suggested behavioral modification and probiotic therapy after PC. Not confirmed in other modalities or at PV.
- *Subject 6*: PC indicated need for intervention; no specific action noted; not confirmed elsewhere.
- *Subject 7*: Independent dentist recommended medication adjustment after PC; not confirmed in other modalities or at PV. The operator could not determine need based on SS, VF, or LC.

### Image and video quality assessment

**Fig. 4:**
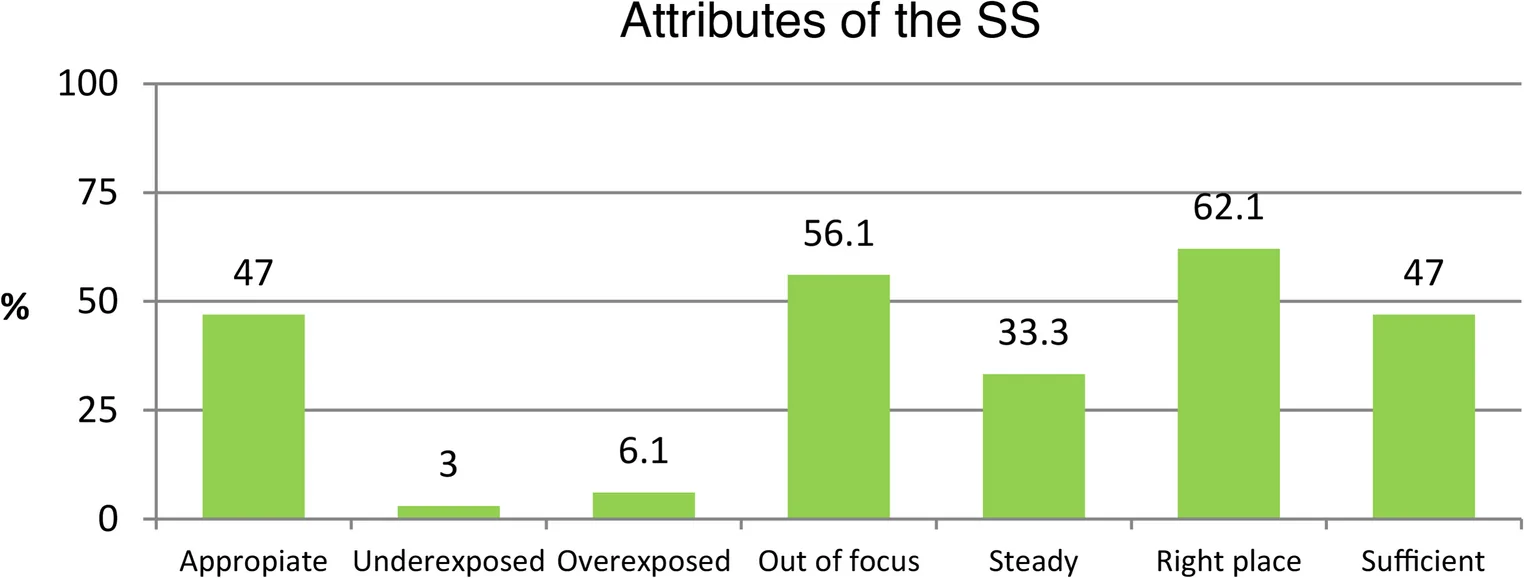
Image quality characteristics of snapshots (SS)

**Fig. 5:**
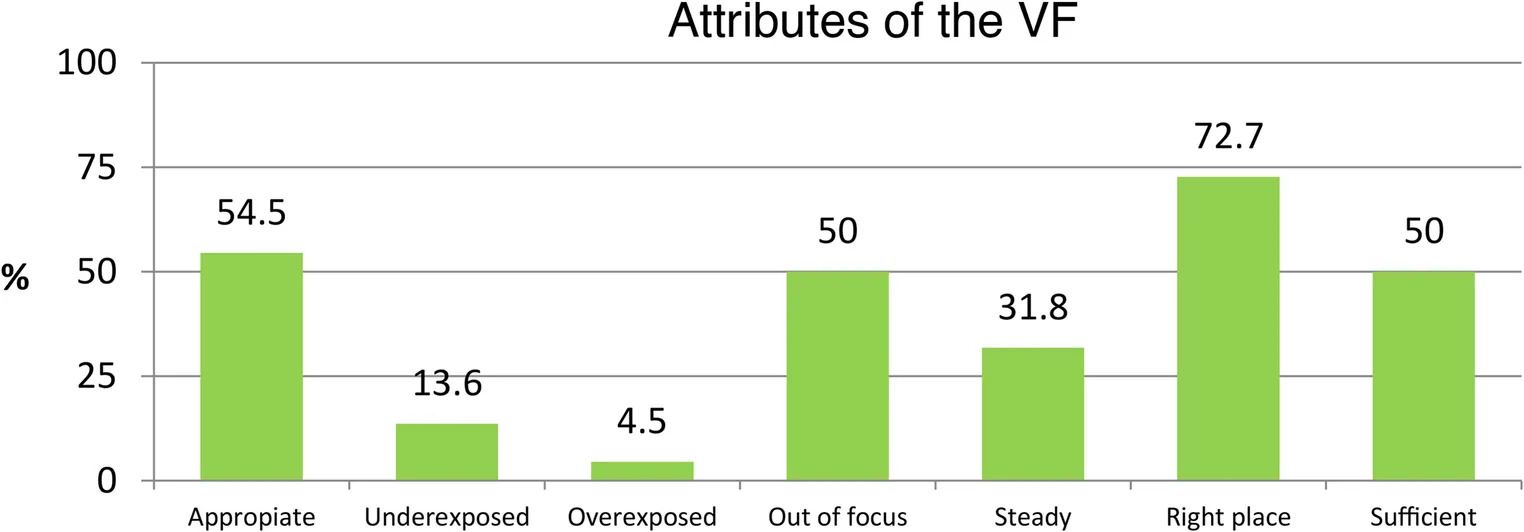
Image quality characteristics of video footage (VF)

**Fig. 6:**
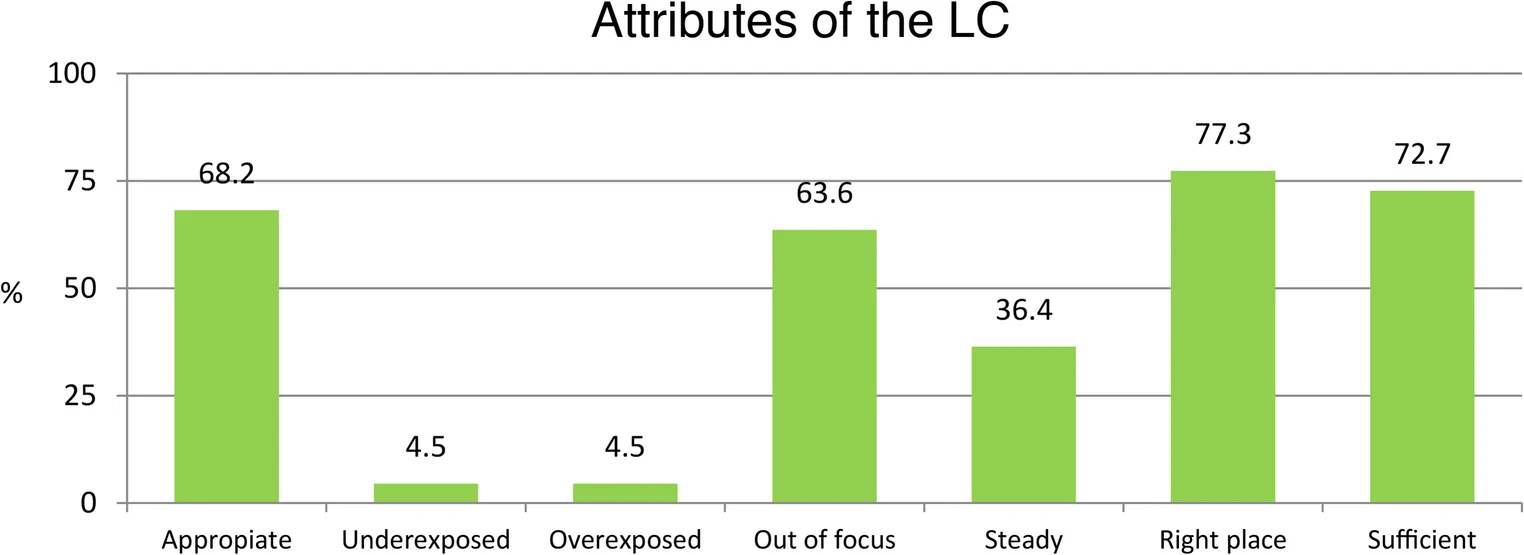
Image quality characteristics of live video call (LC)

A total of 66 snapshots, 22 recorded videos, and 22 live video sessions obtained from 11 participants were independently evaluated by two examiners (Figs. 4, 5 and 6). For patient-generated snapshots, appropriate exposure was observed in 47.0% of images, while 3.0% were underexposed and 6.1% were overexposed. More than half of the images (56.1%) were considered out of focus, and correct intraoral localization was achieved in 62.1% of cases. Overall, 47.0% of the snapshots were deemed diagnostically sufficient.

For patient-generated video footage, appropriate exposure was noted in 54.5% of recordings, whereas 13.6% were underexposed and 4.5% overexposed. Half of the videos (50.0%) were judged to be out of focus. Correct anatomical positioning was achieved in 72.7% of recordings, and 50.0% were considered diagnostically sufficient.

For clinician-guided live video calls, appropriate exposure was achieved in 68.2% of sessions, while both underexposure and overexposure were observed in 4.5% of cases each. A total of 63.6% of sessions were rated as out of focus; however, correct intraoral localization was achieved in 77.3% of cases. Overall, 72.7% of live video calls were considered diagnostically sufficient. Inter-examiner agreement varied among quality parameters.

### Usefulness and preferences

Participants evaluated both the perceived usefulness and their preference for each remote assessment modality. The clinician-guided live call (LC) received the highest preference rating (mean score: 1.68), followed by phone consultation (PC) (2.43), patient-generated snapshots (SS) (2.91), and video footage (VF) (2.98). Preference for PC was significantly higher than for SS (*p* = .009).

In terms of perceived usefulness, LC was rated as the most useful modality in 50% of the assessments, followed by VF, SS, and PC. Although LC demonstrated the greatest advantage compared with the other modalities, these differences did not reach statistical significance.

## Discussion

In this pilot study comparing four remote methods for postoperative wound evaluation after oral surgery, several key findings emerged:

- I) although no subject required an intervention at the physical visit (PV), three patients were incorrectly judged as needing an intervention during remote assessments, mainly during phone consultations and once during a live video call.
- II) using an intraoral camera did not result in more accurate wound assessment than a simple phone consultation.
- III) snapshots-based assessments had the lowest accuracy overall.
- IV) assessments performed by the operating clinician were more closely aligned with the PV findings than those performed by an independent dentist.

Despite the absence of interventions at the PV, remote assessments generated false-positive need for intervention in three cases. This likely reflects the uncertainty clinicians face when they cannot perform a direct examination and must rely on limited or low-quality remote information. In such situations, clinicians may adopt a cautious approach and recommend an intervention “just in case”. While this increases false-positive findings, prioritising patient safety is generally preferable to missing a true complication. Similar findings have been reported in other telemedicine studies [13, 14]. 

The null-hypothesis was not rejected, as intraoral camera–based methods (video footage and live calls) did not consistently outperform phone consultations. However, clinicians preferred live video calls and rated them as the most useful modality. Real-time communication allows clinicians to guide patients, adjust the camera angles and request additional views, which likely explains why live calls yielded the highest percentage of diagnostically adequate images. However, live video calls require both patient and clinician to be available at the same time and depend on a stable internet connection. In contrast, store-and-forward methods such as snapshots or recorded videos offer more flexibility, but cannot be corrected in real time when image quality is suboptimal. A combined approach, using a live video call for initial guidance and store-and-forward methods for follow-up, may offer a practical balance between diagnostic accuracy and feasibility in routine clinical practice.

Snapshot-based assessments the lowest accuracy (66.1%). Still images often suffered from poor focus, inadequate lighting or incorrect angles, limiting reliable wound evaluation. This is a known problem in tele-oral medicine, where patient-generated images often lack sufficient diagnostic clarity [15, 16]. Moreover, wound healing assessments relies on dynamic features such as color, depth, moisture, and surrounding tissues, which may not be fully captured in a single photograph. Future studies should explore guided image capture, better patient instruction and tools to enhance image quality. Combining snapshots with live video assessments may help reduce diagnostic errors and provide a more comprehensive clinical assessment.

Technical factors may also have influenced diagnostic performance. The intraoral camera used in the present study has relatively limited resolution and focus capabilities, which may have contributed to insufficient image quality. In addition, patient-related factors such as shaky hands and limited experience handling intraoral devices likely increased the proportion of diagnostically inadequate or out-of-focus media. Because intraoral cameras are not yet common household devices and correct intraoral positioning is challenging, future approaches may benefit from patient-friendly, smartphone-based solutions. In particular, AI-assisted applications that guide positioning and optimise focus could substantially improve diagnostic adequacy.

Across all remote modalities, assessments performed by the operating clinician agreed more closely with PV findings than those performed by the independent dentist. This may be explained by the operator’s familiarity with the surgical procedure, baseline tissue conditions and expected healing process, which can help with the interpretation of borderline cases. Another possible explanation is that the operator may have been influenced, consciously or or unconsciously by earlier remote assessments. In contrast, the independent dentist, relied exclusively on remote images and videos, without clinical context, making wound healing assessment more difficult, especially when image quality was suboptimal. Similar findings have been reported in telemedicine research, although evidence remains limited [17]. These results suggest that continuity of care may improve diagnostic accuracy in remote postoperative assessments.

Evidence from other surgical fields shows that remote follow-up can be safe and effective and in some settings, has even been associated lower infection rates after hospital discharge [18]. However, findings from chronic disease management such as asthma are mixed and suggest that remote care is not always equivalent to in-person evaluation [19]. Thus, the effectiveness of remote assessment appears to depend on the clinical condition, the quality of the transmitted images and clinician experience. As technology continues to improve, remote postoperative monitoring may become more reliable.

The present findings are broadly consistent with emerging evidence on remote postoperative monitoring across medical and dental disciplines. In a multicentre in the UK. smartphone-based wound assessment following abdominal surgery demonstrated no significant difference in surgical site infection rates compared with standard care, while achieving high patient acceptance and feasibility [20]. Similarly, telephone follow-up after minor dentoalveolar surgery showed no significant differences in complication rates compared with conventional in-person follow-up, with patients expressing a preference for remote care [21]. These observations align with the present findings, in which remote modalities did not differ substantially in clinical outcomes but were perceived as useful by clinicians. Furthermore, digital support tools such as the Exodont App have been shown to improve postoperative medication adherence after minor oral surgery, suggesting that remote technologies may enhance compliance even when diagnostic superiority is not demonstrated [22]. In addition, telesurgical assessment using smartphone messaging and photographic wound evaluation can resolve patient concerns in the majority of cases without requiring in-person review, highlighting the potential of remote systems to reduce unnecessary clinical visits [23]. Taken together, current evidence suggests that while remote assessment may not consistently outperform conventional follow-up in detecting complications, it appears safe, acceptable, and capable of maintaining clinical standards when appropriately implemented.

This study has several limitations. As a pilot investigation, the sample size was small, limiting statistical power to detect clinically meaningful or statistically significant between-group differences and likely contributing to the absence of observed differences across remote assessment modalities. Moreover, the standardized questionnaire used in this study was partially developed ad-hoc specifically for this pilot study investigation and requires further validation in larger studies. Surgical procedures also varied across participants, which may limit generalisability. Importantly, no participant required a true clinical intervention and no major healing complications occurred. This substantially restricts evaluation of the ability of remote methods to detect clinically relevant problems and precludes robust assessment of diagnostic sensitivity for adverse outcomes. Moreover, the same clinician performed both the surgical procedure and the physical visit (PV), which may have introduced bias and favoured the operator’s assessments. Conversely, the operator was also the clinician most familiar with the patient and the intervention performed and such knowledge can enhance clinical interpretation during physical examination. Both aspects should therefore be considered when interpreting the findings.

The use of a brand-specific intraoral camera limits generalizability to other imaging devices. In addition, owing to consistent availability during the study period, an iPhone 8 was used for image review, newer smartphone models with higher screen resolution and improved camera technology may have yielded superior image quality and different diagnostic performance. Finally, feasibility and adoption of remote postoperative assessment in routine clinical workflows depend on adequate patient training, access to intraoral or smartphone-based devices suitable for everyday use and reliable internet connectivity [24]. 

Future studies should include adequately powered sample sizes, standardized surgical procedures, a broader range of healing outcomes and independent examiners for in-person evaluations to better assess diagnostic accuracy and generalizability.

## Conclusion

In this pilot study, smartphone-based intraoral camera methods did not outperform simple phone consultations in identifying postoperative cases requiring intervention after oral surgery. However, remote postoperative wound assessment, particularly live intraoral video evaluation performed by the operating clinician appears to be a feasible and accurate approach. The similar accuracy observed across the four remote modalities for most wound-healing outcomes likely reflects the limited statistical power of this pilot study rather than a true absence of differences between methods. Adequately powered randomised controlled trials are needed to confirm these findings.

## Supplementary Information

Supplementary Material 1.

Supplementary Material 2.

## Data Availability

No datasets were generated or analysed during the current study.
