# Supplementary material for: Smartphone-based patient-driven teledentistry for postoperative monitoring: a pilot study
Source: Clin Oral Investig. 2026 Mar 26;30(4):147. doi: 10.1007/s00784-026-06840-9 (PMC13658371; doi:10.1007/s00784-026-06840-9)
Supplement: Supplementary file 1 [file 784_2026_6840_MOESM1_ESM.docx]

# Appendix

**General wound healing**

General wound healing

%

|  | incorrect | correct | n.a. |
| --- | --- | --- | --- |
| PC | 50 | 40.9 | 9.1 |
| SS | 40.9 | 54.5 | 4.5 |
| VF | 27.3 | 68.2 | 4.5 |
| LC | 36.4 | 59.1 | 4.5 |

**Table 3.1.1.-** **Concordance of General wound healing between modalities**: results of McNemar´s test for testing null hypothesis of balanced discordant proportions (symmetry)

|  | **p-value** |
| --- | --- |
| **PC vs. SS** | 0.727 |
| **PC vs. VF** | 0.125 |
| **PC vs. LC** | 0.453 |
| **SS vs. VF** | 0.250 |
| **SS vs. LC** | 1 |
| **VF vs. LC** | 0.500 |

^*p<0.05; **p<0.01; ***p<0.001^

All modalities provided the same percentage of correct assessments involving the general wound healing.

**Bleeding**

Bleeding

%

|  | incorrect | correct |
| --- | --- | --- |
| PC | 4.5 | 95.5 |
| SS | 18.2 | 81.8 |
| VF | 18.2 | 81.8 |
| LC | 13.6 | 86.4 |

**Table 3.1.2.-** **Concordance of Bleeding between modalities**: results of McNemar´s test for testing null hypothesis of balanced discordant proportions (symmetry)

|  | **p-value** |
| --- | --- |
| **PC vs. SS** | 0.250 |
| **PC vs. VF** | 0.250 |
| **PC vs. LC** | 0.500 |
| **SS vs. VF** | 1 |
| **SS vs. LC** | 1 |
| **VF vs. LC** | 1 |

^*p<0.05; **p<0.01; ***p<0.001^

All modalities provided the same percentage of correct assessments involving this clinical parameter.

**Swelling (IO)**

Swelling (IO)

%

|  | incorrect | correct | n.a. |
| --- | --- | --- | --- |
| PC | 40.9 | 54.5 | 4.5 |
| SS | 13.6 | 68.2 | 18.2 |
| VF | 36.4 | 50 | 13.6 |
| LC | 45.5 | 45.5 | 9 |

**Table 3.1.3.-** **Concordance of Swelling (IO) between modalities**: results of McNemar´s test for testing null hypothesis of balanced discordant proportions (symmetry)

|  | **p-value** |
| --- | --- |
| **PC vs. SS** | 0.250 |
| **PC vs. VF** | 1 |
| **PC vs. LC** | 0.754 |
| **SS vs. VF** | 0.063 |
| **SS vs. LC** | **0.016*** |
| **VF vs. LC** | 0.625 |

^*p<0.05; **p<0.01; ***p<0.001^

Proportion of correct assessments was significantly higher using SS compared to LC (p=0.016).

Moreover, there was a marginal significance showing the superiority of SS against VF (p=0.063).

**Swelling (EO)**

Swelling (EO)

%

|  | incorrect | correct | n.a. |
| --- | --- | --- | --- |
| PC | 9.1 | 90.9 |  |
| LC | 9.1 | 72.7 | 18.2 |

**Table 3.1.4.-** **Concordance of Swelling (EO) between modalities**: results of McNemar´s test for testing null hypothesis of balanced discordant proportions (symmetry)

|  | **p-value** |
| --- | --- |
| **PC vs. SS** | -- |
| **PC vs. VF** | -- |
| **PC vs. LC** | 1 |
| **SS vs. VF** | -- |
| **SS vs. LC** | **--** |
| **VF vs. LC** | -- |

^*p<0.05; **p<0.01; ***p<0.001^

Both modalities provided the same percentage of correct assessments.

**Hematoma (IO)**

Hematoma (IO)

%

|  | incorrect | correct | n.a. |
| --- | --- | --- | --- |
| PC | 22.7 | 59.1 | 18.2 |
| SS | 31.8 | 59.1 | 9.1 |
| VF | 27.3 | 68.2 | 4.5 |
| LC | 22.7 | 72.7 | 4.5 |

**Table 3.1.5.-** **Concordance of Hematoma (IO) between modalities**: results of McNemar´s test for testing null hypothesis of balanced discordant proportions (symmetry)

|  | **p-value** |
| --- | --- |
| **PC vs. SS** | 1 |
| **PC vs. VF** | 1 |
| **PC vs. LC** | 1 |
| **SS vs. VF** | 0.625 |
| **SS vs. LC** | 0.375 |
| **VF vs. LC** | 1 |

^*p<0.05; **p<0.01; ***p<0.001^

All modalities provided the same percentage of correct assessments involving this clinical parameter.

**Hematoma (EO)**

Hematoma (EO)

%

|  | incorrect | correct | n.a. |
| --- | --- | --- | --- |
| PC | 9.1 | 90 |  |
| LC | 9.1 | 81.8 | 9.1 |

**Table 3.1.6.-** **Concordance of Hematoma (EO) between modalities**: results of McNemar´s test for testing null hypothesis of balanced discordant proportions (symmetry)

|  | **p-value** |
| --- | --- |
| **PC vs. SS** | -- |
| **PC vs. VF** | -- |
| **PC vs. LC** | 1 |
| **SS vs. VF** | -- |
| **SS vs. LC** | -- |
| **VF vs. LC** | -- |

^*p<0.05; **p<0.01; ***p<0.001^

Both modalities provided the same percentage of correct assessments.

**Complete flap closure**

Complete flap closure

%

|  | incorrect | correct | n.a. |
| --- | --- | --- | --- |
| SS | 9.1 | 77.3 | 13.6 |
| VF | 9.1 | 77.3 | 13.6 |
| LC | 9.1 | 81.8 | 9.1 |

**Table 3.1.7.-** **Concordance of Complete flap closure between modalities**: results of McNemar´s test for testing null hypothesis of balanced discordant proportions (symmetry)

|  | **p-value** |
| --- | --- |
| **PC vs. SS** | -- |
| **PC vs. VF** | -- |
| **PC vs. LC** | -- |
| **SS vs. VF** | 1 |
| **SS vs. LC** | 1 |
| **VF vs. LC** | 1 |

^*p<0.05; **p<0.01; ***p<0.001^

All three modalities provided the same percentage of correct assessments involving this clinical parameter.

**Incomplete flap closure**

Incomplete flap closure

%

|  | incorrect | correct | n.a. |
| --- | --- | --- | --- |
| SS | 36.4 | 54.5 | 9.1 |
| VF | 40.9 | 54.5 | 4.5 |
| LC | 22.7 | 68.2 | 9.1 |

**Table 3.1.8.-** **Concordance of Incomplete flap closure between modalities**: results of McNemar´s test for testing null hypothesis of balanced discordant proportions (symmetry)

|  | **p-value** |
| --- | --- |
| **PC vs. SS** | -- |
| **PC vs. VF** | -- |
| **PC vs. LC** | -- |
| **SS vs. VF** | 1 |
| **SS vs. LC** | 0.625 |
| **VF vs. LC** | 0.250 |

^*p<0.05; **p<0.01; ***p<0.001^

All three modalities provided the same percentage of correct assessments involving this clinical parameter.

**Fibrin clot**

Fibrin clot

%

|  | incorrect | correct | n.a. |
| --- | --- | --- | --- |
| SS | 36.4 | 54.5 | 9.1 |
| VF | 36.4 | 59.1 | 4.5 |
| LC | 31.8 | 59.1 | 9.1 |

**Table 3.1.9.-** **Concordance of Fibrin clot between modalities**: results of McNemar´s test for testing null hypothesis of balanced discordant proportions (symmetry)

|  | **p-value** |
| --- | --- |
| **PC vs. SS** | -- |
| **PC vs. VF** | -- |
| **PC vs. LC** | -- |
| **SS vs. VF** | 1 |
| **SS vs. LC** | 1 |
| **VF vs. LC** | 1 |

^*p<0.05; **p<0.01; ***p<0.001^

All three modalities provided the same percentage of correct assessments involving this clinical parameter.

**Partial necrosis**

Partial necrosis

%

|  | incorrect | correct | n.a. |
| --- | --- | --- | --- |
| SS | 13.6 | 72.7 | 13.6 |
| VF | 13.6 | 77.3 | 9.1 |
| LC | 9.1 | 81.8 | 9.1 |

**Table 3.1.10.-** **Concordance of Partial necrosis between modalities**: results of McNemar´s test for testing null hypothesis of balanced discordant proportions (symmetry)

|  | **p-value** |
| --- | --- |
| **PC vs. SS** | -- |
| **PC vs. VF** | -- |
| **PC vs. LC** | -- |
| **SS vs. VF** | 1 |
| **SS vs. LC** | 1 |
| **VF vs. LC** | 1 |

^*p<0.05; **p<0.01; ***p<0.001^

All three modalities provided the same percentage of correct assessments involving this clinical parameter.

**Complete necrosis**

Complete necrosis

%

|  | correct | n.a. |
| --- | --- | --- |
| SS | 90.9 | 9.1 |
| VF | 90.9 | 9.1 |
| LC | 90.9 | 9.1 |

**Table 3.1.11.-** **Concordance of Complete necrosis between modalities**: results of McNemar´s test for testing null hypothesis of balanced discordant proportions (symmetry)

|  | **p-value** |
| --- | --- |
| **PC vs. SS** | -- |
| **PC vs. VF** | -- |
| **PC vs. LC** | -- |
| **SS vs. VF** | 1 |
| **SS vs. LC** | 1 |
| **VF vs. LC** | 1 |

^*p<0.05; **p<0.01; ***p<0.001^

All three modalities provided the same percentage of correct assessments involving this clinical parameter.

**Suppuration**

Suppuration

%

|  | correct | n.a. |
| --- | --- | --- |
| SS | 81.8 | 18.2 |
| VF | 90.9 | 9.1 |
| LC | 90.9 | 9.1 |

**Table 3.1.12.-** **Concordance of Suppuration between modalities**: results of McNemar´s test for testing null hypothesis of balanced discordant proportions (symmetry)

|  | **p-value** |
| --- | --- |
| **PC vs. SS** | -- |
| **PC vs. VF** | -- |
| **PC vs. LC** | -- |
| **SS vs. VF** | 1 |
| **SS vs. LC** | 1 |
| **VF vs. LC** | 1 |

^*p<0.05; **p<0.01; ***p<0.001^

All three modalities provided the same percentage of correct assessments involving this clinical parameter.

**Tissue color**

Tissue color

%

|  | incorrect | correct | n.a. |
| --- | --- | --- | --- |
| SS | 54.5 | 45.5 |  |
| VF | 40.9 | 54.5 | 4.5 |
| LC | 27.3 | 63.6 | 9.1 |

**Table 3.1.13.-** **Concordance of Tissue color between modalities**: results of McNemar´s test for testing null hypothesis of balanced discordant proportions (symmetry)

|  | **p-value** |
| --- | --- |
| **PC vs. SS** | -- |
| **PC vs. VF** | -- |
| **PC vs. LC** | -- |
| **SS vs. VF** | 0.500 |
| **SS vs. LC** | 0.125 |
| **VF vs. LC** | 1 |

^*p<0.05; **p<0.01; ***p<0.001^

All three modalities provided the same percentage of correct assessments involving this clinical parameter.

**Need for physical visit before suture removal**

Need for physical visit before suture removal

%

|  | incorrect | correct | n.a. |
| --- | --- | --- | --- |
| PC | 9.1 | 90.9 |  |
| SS | 9.1 | 86.4 | 4.5 |
| VF | 9.1 | 90.9 |  |
| LC | 9.1 | 86.4 | 4.5 |

**Table 3.1.14.-** **Concordance of Need for physical visit between modalities**: results of McNemar´s test for testing null hypothesis of balanced discordant proportions (symmetry)

|  | **p-value** |
| --- | --- |
| **PC vs. SS** | 1 |
| **PC vs. VF** | 1 |
| **PC vs. LC** | 1 |
| **SS vs. VF** | 1 |
| **SS vs. LC** | 1 |
| **VF vs. LC** | 1 |

^*p<0.05; **p<0.01; ***p<0.001^

All modalities provided the same percentage of correct assessments about this question.

**Intervention necessary**

Intervention necessary

%

|  | incorrect | correct | n.a. |
| --- | --- | --- | --- |
| PC | 13.6 | 86.4 |  |
| SS |  | 95.5 | 4.5 |
| VF |  | 95.5 | 4.5 |
| LC | 4.5 | 86.4 | 9.1 |

**Table 3.1.15.-** **Concordance of Intervention necessary between modalities**: results of McNemar´s test for testing null hypothesis of balanced discordant proportions (symmetry)

|  | **p-value** |
| --- | --- |
| **PC vs. SS** | 0.750 |
| **PC vs. VF** | 0.750 |
| **PC vs. LC** | 0.500 |
| **SS vs. VF** | 1 |
| **SS vs. LC** | 1 |
| **VF vs. LC** | 0.750 |

^*p<0.05; **p<0.01; ***p<0.001^

All modalities provided the same percentage of correct assessments about this question.

**Usefulness**

Usefulness

%

|  | most useful | 2nd | 3rd | least useful |
| --- | --- | --- | --- | --- |
| PC | 18.2 | 27.3 | 13.6 | 40.9 |
| SS | 18.2 | 18.2 | 36.4 | 27.3 |
| VF | 13.6 | 36.4 | 27.3 | 22.7 |
| LC | 50 | 13.6 | 31.8 | 4.5 |

**Table 3.1.16.-** **Concordance of Usefulness between modalities**: results of McNemar´s test for testing null hypothesis of balanced discordant proportions (symmetry)

|  | **p-value** |
| --- | --- |
| **PC vs. SS** | 0.340 |
| **PC vs. VF** | 0.227 |
| **PC vs. LC** | 0.142 |
| **SS vs. VF** | 0.267 |
| **SS vs. LC** | 0.156 |
| **VF vs. LC** | 0.099 |

^*p<0.05; **p<0.01; ***p<0.001^

Live call video was the most useful modality for half of 22 assessments. In fact, LC showed the most extreme differences compared to rest of modalities, but not reaching statistical significance.

However, LC was marginally more useful than VF (p=0.099). VF was chosen as the most useful only in 13.6% of assessments.

Additionally, table T35b shows quantitative descriptives for usefulness: mean 2.77 for PC, 2.73 for SS, 2.59 for VF and 1.91 for LC.

**Preference**

Preference

%

|  | most preferred | 2nd | 3rd | least preferred |
| --- | --- | --- | --- | --- |
| PC | 22.7 | 40.9 | 4.5 | 31.8 |
| SS | 18.2 | 13.6 | 27.3 | 40.9 |
| VF |  | 27.3 | 45.5 | 27.3 |
| LC | 59.1 | 18.2 | 18.2 | 4.5 |

**Table 3.1.17.-** **Concordance of Preference between modalities**: results of McNemar´s test for testing null hypothesis of balanced discordant proportions (symmetry)

|  | **p-value** |
| --- | --- |
| **PC vs. SS** | **0.009**** |
| **PC vs. VF** | 0.103 |
| **PC vs. LC** | **0.019*** |
| **SS vs. VF** | **0.036*** |
| **SS vs. LC** | **0.010*** |
| **VF vs. LC** | **0.014*** |

^*p<0.05; **p<0.01; ***p<0.001^

Live call video was the most preferred modality in 59.1% of assessments and significantly higher than the rest.

PC was significantly more preferred than SS (p=0.009).

Distributions of SS and VF were significantly different too (p=0.010). SS was the least preferred in a higher proportion.
