# Supplementary material for: Smartphone-based patient-driven teledentistry for postoperative monitoring: a pilot study
Source: Clin Oral Investig. 2026 Mar 26;30(4):147. doi: 10.1007/s00784-026-06840-9 (PMC13658371; doi:10.1007/s00784-026-06840-9)
Supplement: Supplementary file 2 [file 784_2026_6840_MOESM2_ESM.docx]

*Multi panel figure A*

*Multi panel figure B*

*Multi panel figures A & B - showing parameters 1-17 across all modalities (values on the y-axis are expressed in %)*
